# *Actinomadura graeca* sp. nov.: A novel producer of the macrocyclic antibiotic zelkovamycin

**DOI:** 10.1371/journal.pone.0260413

**Published:** 2021-11-30

**Authors:** Francesco Saverio Tarantini, Mara Brunati, Anna Taravella, Lucia Carrano, Francesco Parenti, Kar Wai Hong, Paul Williams, Kok Gan Chan, Stephan Heeb, Weng C. Chan

**Affiliations:** 1 Biodiscovery Institute, School of Pharmacy, University of Nottingham, University Park, Nottingham, United Kingdom; 2 Fondazione Istituto Insubrico di Ricerca per la Vita (FIIRV), Gerenzano, Italy; 3 Division of Genetics and Molecular Biology, Institute of Biological Sciences, University of Malaya, Kuala Lumpur, Malaysia; 4 Institute of Marine Sciences, Shantou University, Shantou, China; 5 Biodiscovery Institute, School of Life Sciences, University of Nottingham, University Park, Nottingham, United Kingdom; 6 International Genome Centre, Jiangsu University, Zhenjiang, China; Friedrich Schiller University, GERMANY

## Abstract

As part of a screening programme for antibiotic-producing bacteria, a novel *Actinomadura* species was discovered from a soil sample collected in Santorini, Greece. Preliminary 16S rRNA gene sequence comparisons highlighted *Actinomadura macra* as the most similar characterised species. However, whole-genome sequencing revealed an average nucleotide identity (ANI) value of 89% with *A*. *macra*, the highest among related species. Further phenotypic and chemotaxonomic analyses confirmed that the isolate represents a previously uncharacterised species in the genus *Actinomadura*, for which the name *Actinomadura graeca* sp. nov. is proposed (type strain 32-07^T^). The G+C content of *A*. *graeca* 32–07 is 72.36%. The cell wall contains DL-diaminopimelic acid, intracellular sugars are glucose, ribose and galactose, the predominant menaquinone is MK-9(H_6_), the major cellular lipid is phosphatidylinositol and fatty acids consist mainly of hexadecanoic acid. No mycolic acid was detected. Furthermore, *A*. *graeca* 32–07 has been confirmed as a novel producer of the non-ribosomal peptide antibiotic zelkovamycin and we report herein a provisional description of the unique biosynthetic gene cluster.

## Introduction

As originally described [1] and successively revised [2–6], the genus *Actinomadura* defines a variety of aerobic, Gram-positive, non-motile, non-acid-alcohol-fast, chemoorganotrophic soil actinomycetes. These microbes form extensively branched, non-fragmenting substrate mycelium, most often accompanied by aerial hyphae that can maturate into straight, curled, hooked or spiralled spore chains carrying up to 50 arthrospores with a smooth, irregular, rugose, folded, warty or spiny surface. Single spherical spore vesicles may also be produced. Although *Actinomadura* strains are mostly mesophilic and non-pathogenic, some thermophiles have also been identified and strains that are pathogenic to both men and domestic/farm animals [7] are known.

From a chemotaxonomic point of view, *Actinomadurae* are characterised by a type III cell wall, containing both *meso*-2,6-diaminopimelic acid (*meso-*DAP) and *N*-acetylated muramic acid [8], as well as type A1γ peptidoglycan [9]. The plasma membrane contains the non-nitrogenous phospholipids phosphatidylinositol (PI), diphosphatidylglycerol (DPG), phosphatidylglycerol (PG) and a fraction of phosphatidylinositol mannosides (PIMs), in accordance with a type PI lipid composition [10]. Fatty acid tails mainly consist of hexadecanoic acid (C16:0), 14-methylpentadecanoic acid (*iso*-C16:0) and 10-methyloctadecanoic acid (10-meth-C18:0), generally classified as type 3a [11]. Galactose, glucose, mannose, ribose and madurose (3-*O*-methyl-D-galactose) are most abundant within the cell, consistent with a type B sugar composition [8]. Respiratory isoprenoid quinones are predominantly MK-9(H_6_) menaquinones saturated at sites II, III and VIII, with small amounts of MK-9(H_4_) (saturated at sites II and III) and MK-9(H_8_), in line with type 4B2 quinone profile [11]. Mycolic acids are not found in the genus. The G+C composition ranges between 65–73 mol% [7].

As part of a screening programme for antibiotic-producing bacteria, a new microorganism has been isolated from a soil sample collected in Santorini, Greece. The new strain (designated 32–07) was attributed to the genus *Actinomadura* via 16S rRNA analysis and later dereplicated as a novel producer of zelkovamycin, a macrocyclic peptide antibiotic previously found only in *Streptomyces* species [12–15]. The full chemical structure of zelkovamycin was recently established [15]. As part of this work, the chromosomal and plasmid DNA of *Actinomadura* sp. 32–07 has been fully sequenced and the strain subjected to phenotypic, chemotaxonomic and genomic analyses. Furthermore, a bioinformatic approach has been used to identify the genetic cluster coding for the non-ribosomal peptide synthetase system responsible for the biosynthesis of zelkovamycin. Although the list of zelkovamycin-susceptible microorganisms identified to date is fairly limited, the potential strength of this antibiotic lies on its apparent high specificity towards *Staphylococcus aureus* [12, 13]. Particularly interesting would be the potential clinical use of zelkovamycin to treat multi-drug resistant staphylococcal infections.

## Materials and methods

### Strain isolation, maintenance and cultural conditions

Strain 32–07 was isolated from a soil sample collected in Santorini (Greece) (S1 File).

Biomass from mature cultures (typically 14–21 days old) was used to preserve the strain. Agar plates were kept at 4 °C for short-term storage, while liquid cultures in 30% (v/v) glycerol were frozen at -80 °C for long-term storage. Fresh mycelium or glycerol stocks of mycelium and/or spores were routinely grown on ISP medium 1 or 2 and GYEA medium [16, 17].

### Morphological, cultural and physiological properties

Unless stated otherwise, cultures used for morphological, cultural and physiological tests were grown at 28 °C and observations made after 7, 14 and 21 days of growth. Biological media used included: ISP medium 1–7 and 9 [16], glucose-yeast extract agar (GYEA: 15 g/L bacteriological agar, 10 g/L glucose, 10 g/L yeast extract, pH 6.8) [17], yeast extract-iron agar (YEIA: 7.5 g/L bacteriological agar, 3.0 g/L yeast extract, 0.5 g/L ammonium ferric citrate) [18], nutrient agar (NA: 15 g/L bacteriological agar, 5.0 g/L peptone, 3.0 g/L beef extract, pH 7.0) [19], milk agar (50 g/L skim milk powder, 10 g/L bacteriological agar) [20], peptone-salts agar (PSA: 15 g/L bacteriological agar, 10 g/L peptone, 5.0 g/L NaCl, 0.1 g/L CaCI_2_•H_2_O, pH 7.4) [21], urease broth (18 g/L urea, 9.5 g/L Na_2_HPO_4_, 9.1 g/L KH_2_PO_4_, 0.1 g/L yeast extract, 0.01 g/L phenol red, pH 6.8) [22], nitrate broth (5.0 g/L peptone, 3.0 g/L beef extract, 1.0 g/L KNO_3_, pH 7.0) [19] and Tsukamura’s medium (3% glycerol, 20 g/L bacteriological agar, 1.123 g/L trisodium citrate•2H_2_O, 0.5 g/L KH_2_PO_4_, 0.5 g/L MgSO_4_•7H_2_O, pH 7.0) [23].

Morphological observations were made on mature mycelial (14–21 days old) grown on ISP medium 2 or GYEA. For light microscopy, heat-fixed suspensions of mycelia on a glass slide were stained with 0.5% malachite green under constant heating (~90 °C for 5 min), thoroughly washed and briefly counterstained with 0.25% safranin O (30 s). After removing the excess stain, samples were dried and visualised using an Eclipse 50i fluorescence microscope (Nikon) with a Digital Sight DS-Fi1 microscope camera controller (Nikon) and NIS-Elements microscope imaging software (Nikon). For scanning electron microscopy, mycelia were fixed in 3% glutaraldehyde/0.1 M Na-cacodylate buffer (pH 7.2) and post-fixed with 1% OsO_4_. Following dehydration with an EtOH series, samples were dried with hexamethyldisilazane (HMDS) and coated with a 5 nm iridium layer. All samples were imaged using a JEOL 7100F FEG-SEM. Environmental SEM on fixed, uncoated samples was performed using a Quanta650 ESEM (FEI) with a tungsten filament and a GSED or LFD detector.

The pigmentation of the aerial and substrate mycelium of mature cultures was recorded after growth in selected media. Production of melanoid pigments was assessed on ISP medium 1, 6 and 7 after 2, 4 and 21 days of growth.

The ability of the strain to grow at different temperatures (4, 10, 22, 28, 37 and 45 °C), pH (4.0–11.0 in 1.0 increments) and salinity (0–7% in 1% increments) was tested in 96-well plates using GYEA as the basal growth medium.

Growth on sole carbon sources was assessed after 21 days of growth on ISP medium 9 supplemented with 1% (w/v) of myo-inositol, D-mannitol, L-arabinose, D-fructose, D-glucose, L-rhamnose, D-xylose, sucrose, raffinose or cellulose.

Growth on sole nitrogen sources was assessed on Tsukamura’s medium supplemented with 0.1% (w/v) of ammonium sulphate, L-arginine, L-glutamine, glycine, L-histidine, L-leucine, L-methionine, L-phenylalanine or L-valine.

Degradation of aesculin and arbutin (both at 0.1% w/v) was tested in 90 mm Petri dishes using YEIA as the basal growth medium. The development of rich brown pigment (or a deeper colouration than the negative control) around and/or underneath the growth in the test media was recorded as a positive result.

Degradation of elastin (0.3% w/v), hypoxanthine (0.4% w/v), testosterone (0.1% w/v), xanthine (0.4% w/v) and xylan (0.4% w/v) was tested in 90 mm Petri dishes using GYEA as the basal growth medium. The disappearance of the insoluble crystals around and/or underneath the growth in the test media was recorded as a positive result.

Degradation of gelatine (0.4% w/v), starch (1% w/v), tributyrin (1% w/v) and (L)-tyrosine (0.5% w/v) was tested in 90 mm Petri dishes using NA as the basal growth medium. The formation of a clear zone around and/or underneath the growth in the tributyrin medium was recorded as a positive result. The appearance of a clear zone around the growth after flooding the plates with an aqueous solution of 1% tannic acid (for the gelatine medium) or with 95% ethanol (for the starch medium) was recorded as a positive result [19, 24].

Degradation of casein was tested in 90 mm Petri dishes using milk agar as the growth medium. Clearing of the opaque medium around and/or underneath the growth in the test medium was recorded as a positive result.

Degradation of Tween 20, 40, 60 and 80 (all at 1% v/v) was tested in 90 mm Petri dishes using PSA as the basal growth medium. The formation of an opaque halo around and/or underneath the growth in the test media was recorded as a positive result.

Oxidase activity was tested in 90 mm Petri dishes using NA as the growth medium. The mycelial growth was harvested, and the presence of enzymatic activity detected with commercially available oxidase test strips (Honeywell Fluka). The production of a dark purple colour was recorded as a positive result.

Catalase activity was tested in 24-well plates using NA as the growth medium. The evolution of gas from the mycelial growth after flooding the inoculated wells with 3–6% H_2_O_2_ was recorded as a positive result [25].

Urease activity was tested in 5 mL test tubes using urease broth as the growth medium. The production of red colour in the test medium was recorded as a positive result [22].

H_2_S production was tested in 15 mL slant tubes using ISP medium 6 as the growth medium. A commercial H_2_S test strip (Sigma) was inserted between the cap and the tube throughout the incubation period. Blackening of the strip or the test medium was recorded as a positive result [25].

Nitrate reduction was tested in 50 mL tubes using nitrate broth as the growth medium. The content of nitrate (NO_3_^-^) and nitrite (NO_2_^-^) was measured using a commercial nitrate/nitrite test stick (Quantofix) and an increase of the nitrite concentration in the test medium was recorded as a positive result.

Antibiotic susceptibility testing was performed by the DSMZ Identification Service (Braunschweig, Germany) according to the standard DIN disc diffusion method [26], using 5, 10, 15, 20, 30, 40, 50, 75, 100 or 300 μg of antibiotic per disc.

### Chemotaxonomic characteristics

The analysis of chemotaxonomic features was carried out by the DSMZ Identification Service (Braunschweig, Germany) according to the procedures outlined below.

Cellular fatty acids were saponified, methylated and extracted from an actively growing culture using minor modifications of the methods of Miller and Kuykendall [27, 28] and a Sherlock Microbial Identification System (MIS) (MIDI Inc., Newark, USA) consisting of an Agilent 6890N gas chromatograph fitted with a 5% phenylmethyl silicone capillary column (0.2 mm x 25 m), a flame ionization detector and an Agilent 7683A automatic sampler. Data were analysed with MIS Standard Software against a proprietary database [29], (MIDI Inc., Newark, USA).

Respiratory quinones and lipoquinones were extracted from freeze-dried cells using the two-stage method described by Tindall [30, 31]. Menaquinones were separated by TLC on silica gel and further analysed by RP-HPLC on an LDC Analytical system (Thermo Separation Products) fitted with a reverse-phase C18 column (Macherey-Nagel, 2 mm x 125 mm, 3 μm, RP-18) and the effluent monitored at 269 nm.

Polar lipids were extracted from freeze-dried biomass (after lipoquinones extraction) using a modification of the method of Bligh and Dyer [32] and separated by two-dimensional TLC on silica gel. Total lipid material was detected as described by Tindall [33].

The presence of 2,6-diaminopimelic acid (DAP) isomers or OH-DAP in whole-cell hydrolysates was determined by TLC on cellulose plates using the solvent system of Rhuland [34].

Whole-cell sugars from cell hydrolysates were analysed by TLC on cellulose plates according to Staneck and Roberts [35].

Mycolic acids were extracted and analysed as described elsewhere [36].

### Genomic and phylogenetic analyses

The genomic DNA of *Actinomadura* sp. 32–07 was extracted using Zymo Research Soil Microbe DNA Kit (Zymo Research, USA). Subsequently, the purity and quantity of the extracted genomic DNA were measured using NanoDrop 2000 spectrophotometer (Thermo Fisher Scientific, Waltham, MA, USA) and Qubit 2.0 fluorometer (Thermo Fisher Scientific, Waltham, MA, USA), respectively [37]. A 20-kb template library was prepared using the BluePippin size-selection system and the full genome is sequenced using a Pacific Biosciences RS II platform with P5-C3 chemistry following the MagBead loading protocol (Pacific Biosciences, Menlo Park, CA) [38]. The average sequencing coverage is 122.82 times. De novo assembly of the genome was performed using Hierarchical Genome Assembly Process (HGAP) version 2.0 [39]. The circularity of the assembled genome was investigated using Contiguity [40] and the overlapping ends were trimmed using Minimus2 of the AMOS software package [41]. The sequenced genome was annotated using Prokka 1.11 [42].

Genome-based identification of strain 32–07 was performed with EZBioCloud’s TrueBac^™^ service [43] using the complete sequence of *Actinomadura* sp. 32–07. The average nucleotide identity (ANI) and the similarity values of selected genes for the 15 most similar species present in the database were returned as part of the results.

For the phylogenetic analysis, the 16S rRNA gene consensus of strain 32–07 was compared with the homologous sequence from type strains of *Actinomadura* species with validly published names as of July 2019 [44, 45] obtained from the SILVA database [46]. The sequences were aligned using MUSCLE [47, 48] and the alignment was manually verified and adjusted prior to the construction of phylogenetic trees in MEGA X [49] using the neighbour-joining (NJ), maximum likelihood (ML) and maximum parsimony (MP) methods [50–52]. For the NJ method, the evolutionary distances were computed using the p-distance method. For the ML analysis, the Tamura-Nei model [53] was used in conjunction with discrete Gamma distribution (+G, 5 categories, parameter = 0.3769) and assuming evolutionarily invariable sites (+I, 39.89% of sites). Initial tree(s) for the heuristic search were obtained automatically by applying Neighbor-Join and BioNJ algorithms to a matrix of pairwise distances estimated using the Maximum Composite Likelihood (MCL) approach, and then selecting the topology with superior log likelihood value. The MP tree was obtained using the Tree-Bisection-Regrafting (TBR) algorithm with search level 3, in which the initial trees were obtained by the random addition of sequences (10 replicates). For all trees, positions with less than 80% site coverage were eliminated before the analysis (partial deletion option) and the stability of the clades was appraised by bootstrap analysis with 1000 replicates [54]. When available, *Actinomadura* sequences of the RNA polymerase subunit genes *rpoB*/*rpoC* were collected and analysed in the same way.

Prediction of secondary metabolite biosynthetic clusters was performed on the complete genome using antiSMASH 5.0 [55]. The results were manually inspected and a candidate cluster matching the putative arrangement of zelkovamycin synthetase (inferred from the chemical structure of zelkovamycin) was selected.

### Fermentation, extraction and detection of zelkovamycin

For the fermentative production of zelkovamycin, mature growth from a single GYEA plate was used to start an inoculum culture in 100 mL of seed broth (18 g/L dextrose, 8 g/L soytone, 4 g/L CaCO_3_, 2 g/L yeast extract, 1 g/L NaCl, pH 7.3) and incubated with shaking (200 RPM) for 5 days at 30 °C. Biomass from the seed broth was harvested, triturated and used to inoculate 100 mL of the fermentation broth (20 g/L soluble starch, 8 g/L casamino acids, 3 g/L CaCO_3_, 2 g/L proteose peptone No. 3, 2 g/L yeast extract, 1 g/L dextrose). Fermentation was carried out with shaking (200 RPM) for 10 days at 30 °C.

Biomass was harvested from the fermentation broth by centrifugation and zelkovamycin was extracted from the cleared medium using Diaion HP-20 polystyrene resin (Sigma-Aldrich) at a ratio of 1 g per 40 mL. After 2 h of incubation at room temperature with gentle shaking (200 RPM), the resin was recovered and loaded into a 10-mL disposable PD-10 chromatography column (GE Healthcare) and stepwise elution performed with increasing concentrations of methanol.

The eluates were analysed by LC-MS on an Accela modular system (ThermoScientific, Hemel Hempstead, UK) equipped with a Gemini C18 2μ (100 x 3 mm) chromatography column (Phenomenex). Stepwise elution was performed with increasing concentrations of acetonitrile in 0.1% aqueous formic acid. Mass spectra were collected using an Exactive orbital ion-trap mass spectrometer (ThermoScientific, Hemel Hempstead, UK) over a run time of 15 min with an m/z acquisition range of 150–1000. Commercially available zelkovamycin (AdipoGen) was used as a standard for the optimisation of the detection method, and the characteristic ion-mass signal of zelkovamycin was used in all subsequent analyses.

### Antimicrobial susceptibility testing

The *in vitro* antimicrobial activity of commercial zelkovamycin (AdipoGen) and zelkovamycin extracts from fermentation broth was determined by applying test samples to antimicrobial susceptibility test discs (Oxoid) and using *Staphylococcus aureus* SH1000, according to the EUCAST disk diffusion method [56].

## Results and discussion

As part of a screening campaign to identify novel producers of antibacterial compounds, the broth extract of the microorganism ‘(ID) 32–07’ was initially found to display potent anti-*S*. *aureus* activity. Following routine HPLC separation, and MS ([M+H]^+^ = 780.846, [M+NH_4_]^+^ = 797.89; [M+Na]^+^ = 802.90) and UV spectrum (a major peak at 227 nm and a shoulder at 291 nm) analyses of a single fraction, the anti-*S*. *aureus* activity was ascribed to the macrocyclic peptide, zelkovamycin. Preliminary 16S rRNA analysis of the producer strain suggested it is a previously uncharacterised *Actinomadura* species.

In order to elucidate the taxonomic position of isolate 32–07, a genomic and phylogenetic evaluation was initially performed on its complete genome sequence. A polyphasic approach consisting of morphological, cultural, physiological and chemotaxonomic analyses was then applied to compare the strain to its phylogenetically closest relative.

### Genomic and phylogenetic analyses

The annotated sequences for the whole chromosomal and plasmid DNA have been deposited in the GenBank database under accession numbers CP059572 (chromosome), CP059573 (plasmid 1) and CP059574 (plasmid 2).

The *in silico* genome analysis of isolate 32–07 identified it as an *Actinomadura* species. Based on average nucleotide identity (ANI) over whole genomes, the most similar species present in the database was *Actinomadura macra* NBRC 14102^T^ [57] (89.61%), followed by *Actinomadura fibrosa* (86.70%), *Actinomadura roseirufra* (86.66%), *Actinomadura formosensis* (86.23%), *Actinomadura nitritigenes* (86.22%) and *Actinomadura geliboluensis* (86.18%) (Table 1). All values were well below the accepted 95–96% threshold for species circumscription [58], suggesting that the novel isolate constitute a phylogenetically-separate species.

**Table 1:** Whole-genome comparison between *Actinomadura* sp. 32–07 and closely related *Actinomadura* species identified by TrueBac^™^.

	*A*. *macra*	*A*. *fibrosa*	*A*. *roseirufra*	*A*. *formosensis*	*A*. *nitritigenes*	*A*. *geliboluensis*
	NBRC 14102^T^					
ANI	89.61%	86.70%	86.66%	86.23%	86.22%	86.18%
ANI coverage	70.7%	37.70%	41.0%	35.20%	32.27%	32.0%
16S similarity	99.03%	98.25%	98.61%	98.33%	98.03%	98.54%
*recA* similarity	91.69%	N/A	93.28%	94.20%	N/A	74.14%
*rplC* similarity	100%	100%	N/A	96.47%	96.47%	96.47%

All ANI values fall below the designated threshold for species circumscription (95–96%). N/A = not available.

**Fig 1:**
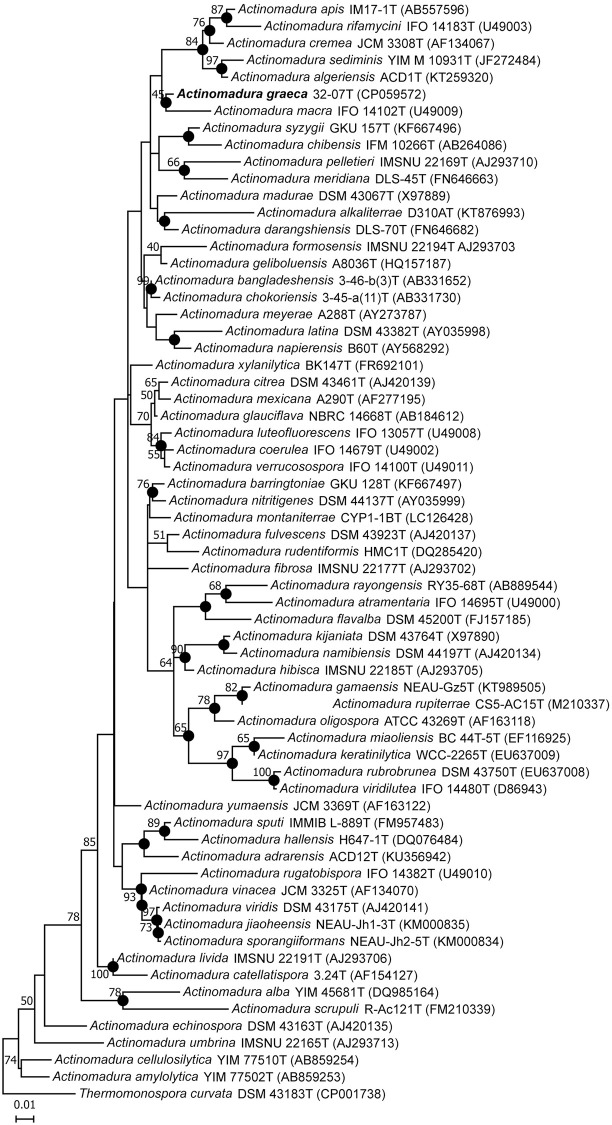
Maximum likelihood tree generated from nearly complete 16S rRNA gene sequences showing the relationship between *Actinomadura* sp. 32–07 and the type strains of species with validly published names in the genus *Actinomadura*. *Thermomonospora curvata* DSM 43183^T^ was used as an outgroup. The tree with the highest log likelihood is shown. The percentage of replicate trees in which the associated taxa clustered together in the bootstrap test (1000 replicates) is shown next to the branches (only values above 40% are shown). The tree is drawn to scale, with branch lengths measured in number of substitutions per site (bar = 0.01 substitutions per site). Nodes marked with a full circle were also recovered in the neighbour-joining and maximum parsimony trees. T’s at the end of strain names indicate type strains.

The phylogenetic position of *Actinomadura* sp. 32–07 was further investigated by obtaining a multiple alignment between its 16S rRNA gene sequence and that of other species in the genus *Actinomadura*, from which NJ, ML and MP trees were reconstructed. In all three cases, the strain formed a clade with *A*. *macra* NBRC 14102^T^, supporting the existence of a close evolutionary relationship between the two, albeit the low number of occurrences among all iterations of each tree reflected their somewhat loose similarity (Fig 1). An equivalent analysis with the *rpoB*/*rpoC* sequences, when these are available, produced the same result (S1 Fig).

### Morphological, cultural and physiological properties

**Fig 2:**
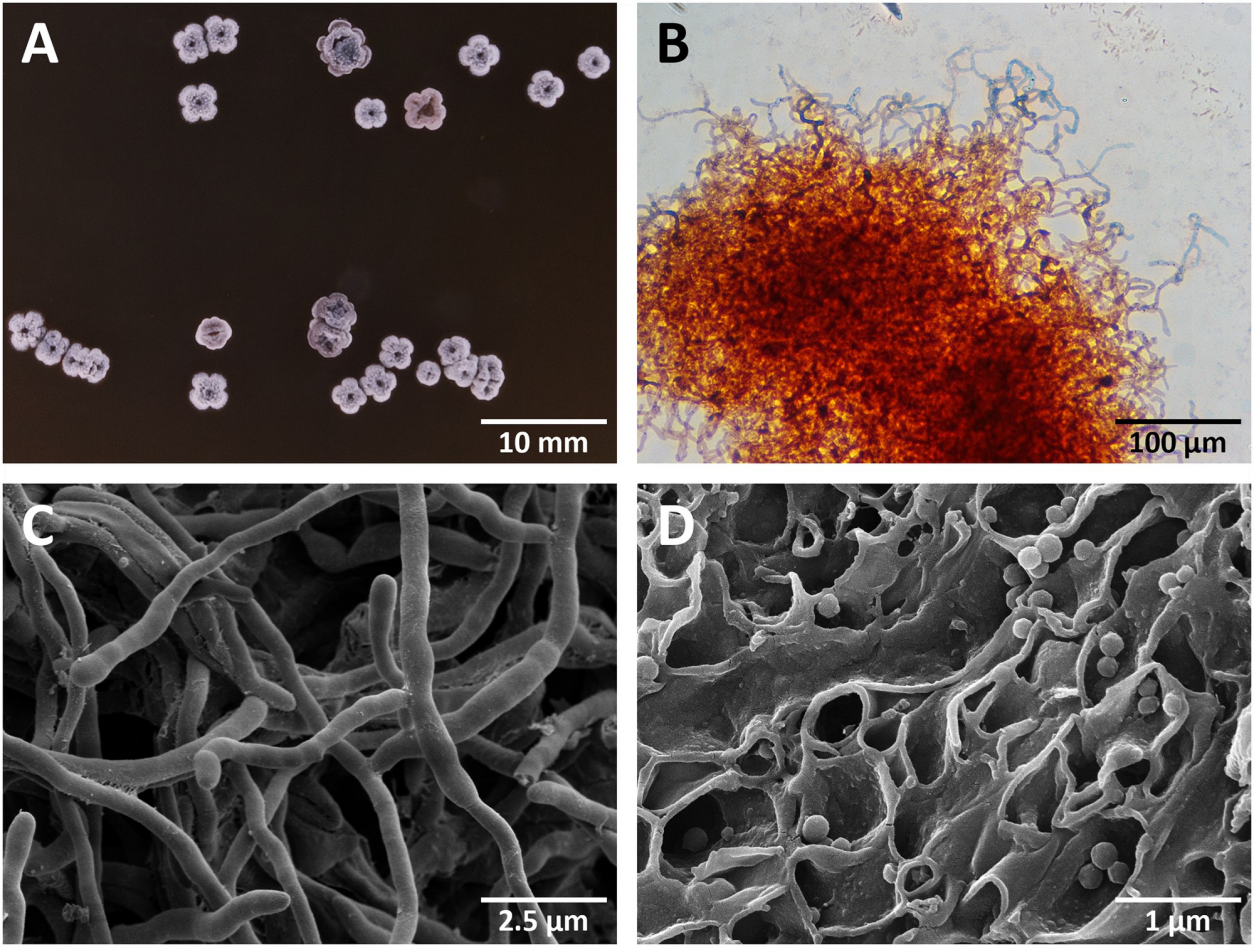
Photographic, light microscopy and electron microscopy images of *Actinomadura* sp. 32–07. After 21 days of growth on ISP medium 2, colonies are characterised by four major lobes, which often split in two minor sectors, and a central hollow pit (A, no magnification). Spore chains appear as long, flexuous series of subtle oval swelling stemming from a thinner, smooth hypha. Endospores inside the swellings can be viewed by light microscopy after staining with malachite green and counterstaining with safranin O (B, 100X magnification) or by closer inspection with electron microscopy (C, 8,500X magnification). Free spores of *Actinomadura* sp. 32–07 appear spherical and smooth, measuring 200–300 nm in diameter (D, 20,000X magnification).

After 5–7 days of incubation on ISP medium 2, strain 32–07 formed small, flat, spherical colonies that subsequently rapidly increased in size, reaching up to 1 mm in height and 2 mm in width after 21 days of growth. Mature colonies developed a multi-lobate toroidal shape characterised by four major lobes splitting in two minor sectors around a central hollow pit (Fig 2A).

Under microscopic observation, colonies revealed an extensively branching substrate mycelium carrying aerial hyphae with an average diameter of 0.5 μm, which often differentiated into long, flexuous spore chains appearing as a series of subtle oval swellings stemming from an otherwise similar filament. Single round terminal swellings were sometimes also observed. As the presence of spores in spore chains was not always obvious by scanning electron microscopy (SEM), a combination of light microscopy and endospore staining was employed. This method allowed the detection of an average of 10–20 spores per chain (Fig 2B and 2C). Once released from the hyphae, spores appeared spherical and smooth under SEM and measured 0.2–0.3 μm in diameter (Fig 2D).

Growth on agar media was generally good on GYEA, NA and ISP medium 2, moderate on ISP 3 and poor on IPS 4, 5, 6, 7 and Tsukamura’s medium. The coloration of the aerial mycelium varied from white to grey on ISP medium 2, pink to white and grey on ISP 3, yellow to white on ISP 4, dark green to dark brown on ISP 5, pink to white on ISP 6, brown on ISP 7 and ISP 9 supplemented with glucose or sucrose, yellow to brown on GYEA, white with pinkish on NA and dark green to dark brown on Tsukamura’s medium. The substrate mycelium was dark brown on ISP medium 2, pink on ISP 3, white on ISP 4, yellow-brown with green on ISP 5, pink on ISP 6, brown on ISP 7, yellow-brown with orange on GYEA or ISP 9 supplemented with glucose, yellow-brown on ISP 9 supplemented with sucrose, orange with pink on NA and dark grey on Tsukamura’s medium.

When treated with aqueous acid (0.05 M HCl), the substrate mycelium of cultures grown on ISP medium 2, 3 and 9 (both with glucose and sucrose) lost its colouration to different extent. Under basic conditions (0.05 M NaOH), mild to intense discolouration was also observed on ISP 3, 5 and 9 (both with glucose and sucrose). Diffusible melanoid pigments were not produced on ISP medium 6 and 7 or in any other tested media, but soluble colours other than brown or black could be observed on ISP 1 (light yellow), GYEA (orange, discolouring at basic pH) and Tsukamura’s medium (dark yellow).

The physiological properties of *Actinomadura* sp. 32–07 included the utilisation of D-glucose and sucrose as sole carbon sources on ISP medium 9, but not *myo*-inositol, D-mannitol, L-rhamnose, raffinose or cellulose (utilisation of L-arabinose, D-fructose and D-xylose was uncertain). On Tsukamura’s medium, none of the tested nitrogen sources (ammonium sulphate, arginine, glutamine, glycine, histidine, leucine, methionine, phenylalanine and valine) was found to produce abundant growth by itself, although darkening of the pre-existing mycelial inoculum became evident in most cases (particularly with phenylalanine and valine) and colouration of the medium was observed in all inoculated media (including the negative control, lacking any nitrogen source). On GYEA, strain 32–07 was able to grow between 28 and 37 °C, showed a pH optimum of 6.0–8.0 and could tolerate up to 3% (w/v) of NaCl. The metabolic and enzymatic capabilities of the isolate were assessed. Positive tests included degradation of aesculin, arbutin, hypoxanthine, gelatine, starch, L-tyrosine, casein, Tween 20, Tween 40, Tween 60 and Tween 80, as well as catalase activity, H_2_S production and nitrate reduction. On the other hand, the strain was negative for the degradation of elastin, xanthine, xylan and tributyrin, as well as for oxidase and urease activity.

The most effective antibiotics (diameter of inhibition ≥50 mm) were bacitracin, imipenem, linezolid, ticarcillin and clindamycin, but appreciable sensitivity (diameter of inhibition 40–44 mm) to ampicillin, piperacillin, ceftriaxone, moxifloxacin, and chloramphenicol was also detected. Neomycin was shown to have lesser effect (diameter of inhibition < 36 mm). *Actinomadura* sp. 32–07 was found to be resistant to aztreonam, norfloxacin, nitrofurantoin and fosfomycin.

A list of the morphological, cultural and physiological properties that differentiate *Actinomadura* sp. 32–07 from *A*. *macra* NBRC 14102^T^ is presented in Table 2.

**Table 2:** Main morphological, cultural and physiological traits that distinguish *Actinomadura* sp. 32–07 from *A*. *macra* NBRC 14102^T^.

	*Actinomadura* sp. 32–07	*A*. *macra* NBRC 14102^T^
**MORPHOLOGICAL FEATURES**		
Sporulation	Yes, not evident	Yes, scarce
Spore chains	Flexuous	Straight, flexuous, hooked or coiled
Spore number	10–20	4–15
Spore morphology	Spherical, smooth	Oval or elliptical, smooth
Spore dimensions	0.2–0.3 μm	0.8–1.0 x 1.2–2.0 μm
**CULTURAL FEATURES**		
ISP 2		
Growth	Good	Good
Aerial mycelium	White to grey	Grey to greyish
Substrate mycelium	Dark brown	Black
Soluble pigments	None	Brown
ISP 3		
Growth	Moderate	Moderate
Aerial mycelium	Pink to white	Cream to faint pink
Substrate mycelium	Pink	Cream to faint pink
Soluble pigments	None	None
ISP 4		
Growth	Poor	Very scant
Aerial mycelium	Yellow to white	Colourless or pale greyish
Substrate mycelium	White	Colourless
Soluble pigments	None	None
ISP 5		
Growth	Poor	Poor
Aerial mycelium	Dark green to dark brown	Cream
Substrate mycelium	Yellow-brown to green	Cream
Soluble pigments	None	None
Nutrient agar		
Growth	Good	Poor to moderate
Aerial mycelium	White to pinkish	Cream
Substrate mycelium	Orange to pink	Cream
Soluble pigments	None	None
Nutrient agar + gelatine		
Growth	Very good	Moderate
Aerial mycelium	Light pink and white, grey dots	Avellaneous
Substrate mycelium	Dark pink	Avellaneous
Soluble pigments	Faint yellow	None
Nutrient agar + starch		
Growth	Very good	Moderate
Aerial mycelium	Light pink and white, grey dots	Avellaneous
Substrate mycelium	Light red	Avellaneous
Soluble pigments	Faint yellow	None
Nutrient agar + tyrosine		
Growth	Very good	Poor
Aerial mycelium	Light pink, grey dots	Cream to pale yellowish
Substrate mycelium	Light red, black dots	Cream to pale yellowish
Soluble pigments	Faint brown	None
Milk agar		
Growth	Poor	None or few colonies
Aerial mycelium	Dark purple to light red	Pale pinkish orange
Substrate mycelium	Dark purple to light red	Pale pinkish orange
Soluble pigments	Dark green or blue (around growth)	None
	Dark yellow (medium)	
Yeast-starch agar		
Growth	Poor	Moderate
Aerial mycelium	Grey	Pinkish orange
Substrate mycelium	Dark green and pink	Pinkish orange
Soluble pigments	None	Pale yellow
Jensen isolation agar		
Growth	Poor	Poor to moderate
Aerial mycelium	Light brown with pink and dark purple	Colourless to cream
Substrate mycelium	Light brown with pink and dark purple	Colourless to cream
Soluble pigments	Faint yellow or light green	None
Czapek’s agar		
Growth	Poor	Poor
Aerial mycelium	Brown to light brown	Pale cream
Substrate mycelium	Brown to light brown	Colourless
Soluble pigments	Light brown	None
Emerson’s agar		
Growth	Poor	Moderate to good
Aerial mycelium	Light red with dark purple	Greyish, black
Substrate mycelium	Pink with light brown	Greyish, black
Soluble pigments	Yellow	None
Water agar		
Growth	Scant	Scant
Aerial mycelium	Colourless	Colourless
Substrate mycelium	Colourless	Colourless
Soluble pigments	None	None
**PHYSIOLOGICAL FEATURES**		
Utilisation of sole carbon sources (1.0%, w/v):		
L-Arabinose	±	-
D-Glucose	++	+
Utilisation of sole nitrogen sources (0.1%, w/v):		
L-Leucine	-	+ ^‡^
L-Phenylalanine	-	+ ^‡^
L-Valine	-	+ ^‡^
Temperature tolerance:		
10 °C	-	+ ^‡^
22 °C	-	+
45 °C	-	- / + ^‡^
pH tolerance:		
pH 9.0	-	+ ^‡^
Degradation of:		
Casein	+	- / + ^‡^
Elastin	-	+ ^‡^
Hypoxanthine	+	- ^‡^
Starch	+	-
Tributyrin	-	+ ^‡^
Tween 40	±	+ ^‡^
Tyrosine	+	+ / − ^‡^
Resistance to inhibitory compounds:		
Ampicillin	-	+ ^‡^
Neomycin	-	- (above 3 μg/mL) ^‡^

Unless indicated otherwise, data for the reference strain of *A*. *macra* was taken from the original description of the species [57]. Growth on single carbon sources was rated as follows: “++” equal or greater than the positive control, “+” better than negative control but less than the positive control, “±” only slightly better than the negative control, “-”similar or less than the negative control.

^‡^: Data from Trujillo and Goodfellow [59].

### Chemotaxonomic characteristics

The chemotaxonomic markers of *Actinomadura* sp. 32–07 were in line with those of other members of the genus *Actinomadura* (Table 3). The DL-2,6-diaminopimelic acid (DL-DAP) was detected as the cross-linking amino acid of peptidoglycan, while no mycolic acids were found. Phosphatidylinositol (PI), diphosphatidylglycerol (DPG) and phosphoglycolipids (PGL) were the predominant phospholipids in the plasma membrane and mostly contained fatty acids, such as hexadecanoic acid (palmitic acid, C16:0), *cis*-9-octadecenoic acid (oleic acid, C18:1 ω9c), 10-methyloctadecanoic acid (tuberculostearic acid, 10-methyl-C18:0) and *cis*-9-hexadecenoic acid (palmitoleic acid, C16:1 ω9c). The predominant menaquinone was MK-9(H_6_), with minor amounts of MK-9(H_4_), MK-9(H_8_) and MK-9(H_2_). Galactose, glucose and ribose were the main sugars found in the cell.

**Table 3:** Summary of the main chemotaxonomic markers of *Actinomadura* sp. 32–07.

Chemotaxonomic markers	Type and quantity	
**Isomer of DAP**	DL-DAP	
**Mycolic acids**	Absent	
**Phospholipids**	PI, DPG, PGL	
**Fatty acids**		
* Saturated*	C14:0	2.44%
	C15:0	2.04%
	C16:0	41.07%
	C17:0	3.37%
	C18:0	2.68%
* Unsaturated*	C16:1 ω9c	10.42%
	C17:1 ω9c	3.44%
	C18:1 ω9c	16.54%
* Branched*	*iso-*C16:0	3.63%
* Methylated*	10-methyl-C16:0	1.07%
	10-methyl-C17:0	1.22%
	10-methyl-C18:0	10.50%
**Menaquinones**	MK-9(H_6_)	64%
	MK-9(H_4_)	20%
	MK-9(H_8_)	12%
	MK-9(H_2_)	3%
**Whole-cell sugars**	Galactose, glucose, ribose	

DL-2,6-diaminopimelic acid (DL-DAP), but no mycolic acids, are present in the cell wall. Phosphatidylinositol (PI), diphosphatidylglycerol (DPG) and phosphoglycolipid (PGL) are the main phospholipids, with palmitic acid as the predominant fatty acid. The menaquinone system is mainly composed of MK-9(H6). Galactose, glucose and ribose are the main sugars. Fatty acids that contributed to less than 1% of the total are omitted.

### Identification of the zelkovamycin gene cluster

**Fig 3:**
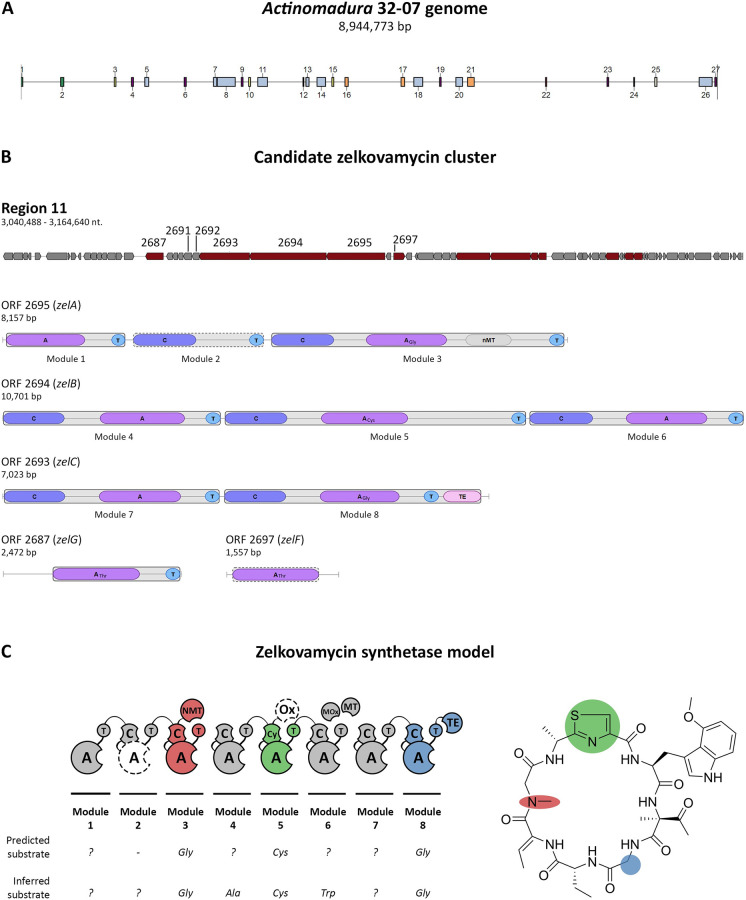
Selected results of the antiSMASH analysis on the genome of *Actinomadura* sp. 32–07. (A) A total of 27 regions were identified that contained one or more predicted biosynthetic clusters. (B) Of all the results, only region 11 (124,153 bp) contained a gene cluster coding for NRPS modules whose arrangement and domain composition agreed with the chemical structure of zelkovamycin (*zelA–C*). The core biosynthetic clusters in the region are highlighted in red and ORFs are numbered according to their position in the genome. Where available, the modules and domains coded by the ORFs of interest and their predicted substrate specificity are shown. (C) Schematic of the organization of the putative zelkovamycin synthetase according to the structure of zelkovamycin. The similarities between the expected residue specificity of some A domains and the respective software prediction are illustrated, along with the domains missing from the software prediction.

The genome-mining software analysis performed on the chromosome of *Actinomadura* sp. 32–07 for the detection of genes involved in the production of secondary metabolites uncovered the existence of at least 27 separate regions containing, among others, biosynthetic gene clusters ascribed to terpene synthases, lanthipeptide synthases, type I and II polyketide synthases (T1PKS and T2PKS) and non-ribosomal peptide synthetases (NRPS), and hybrids thereof (Fig 3A). Some of these have high sequence similarity with gene clusters know to direct the biosynthesis of secondary metabolites, such as ectoine (100% similarity), SapB (75%), nanchangmycin (69%), DADH (51%), neoabyssomicin/ abyssomicin (50%), venezuelin (50%), hopene (50%) and 2-methylisoborneol (50%).

The best match for a candidate zelkovamycin biosynthetic gene cluster was identified in a ~124-kbp region containing several contiguous ORFs predicted to code for complete NRPS, T1PKS and T2PKS modules as well as several stand-alone domains. Specifically, three biosynthetic genes in the region, hereby named *zelA* (ORF 2695), *zelB* (ORF 2694) and *zelC* (ORF 2693), were found to encode for eight NRPS modules whose arrangement, domain composition and substrate specificity agreed with the chemical structure and composition of zelkovamycin (Fig 3B and 3C). Some key similarities included a glycine-specific adenylation (A) domain in conjunction with an *N-*methylation (NMT) domain for the incorporation of *N-*methylglycine, an heterocyclisation (Cy) domain in conjunction with a cysteine-specific A domain for the formation of a thiazoline ring, and a glycine-specific A domain for the incorporation of glycine. As with most NRPS systems, a putative initiation module lacking a condensation (C) domain and a termination module featuring a C-terminal thioesterase (TE) domain were also present at opposing ends of the cluster.

On the other hand, some important domains and enzymes expected to take part in the biosynthesis of zelkovamycin were not automatically included in the software prediction, namely the A domain in module 2, an oxidase (Ox) domain in module 5 (required for the oxidation of the thiazoline ring into a thiazole heterocycle) and many of the stand-alone or post-synthetic enzymes required for the modification or decoration of the zelkovamycin precursor. However, additional BLAST and conserved domain prediction analyses on the translated DNA sequence of modules 2 and 5 revealed the existence of selected regions with appreciable homology to amino acid adenylation domains (TIGR01733) and mcbC-like oxidoreductase domains (cd02142), respectively, leading to the hypothesis that the corresponding enzymatic activities may still be provided in some form by each module.

A similar analysis on two genes immediately downstream of *zelC* (ORFs 2692 and 2691, Fig 3B) revealed that the translated nucleotide sequence of ORF 2691 (*zelE*) has appreciable homology to the tryptophan 2,3-dioxygenase superfamily (TIGR03036), members of which have been previously found and characterised in *Amycolatopsis* and *Streptomyces* species (GenBank SEF24281.1 and PKV74255.1). The product of ORF 2692 (*zelD*) instead showed sequence homology to enzymes in the superfamily of *S*-adenosylmethionine-dependent methyltransferases (SAM-MT), some of which specifically target oxygen atoms (*O*-MT) and have been previously characterised in *Streptomyces* and *Nonomuraea* species (GenBank SFT14077.1, WP_016573776.1 and AIW58898.1). It is therefore postulated that ZelD and ZelE may be involved in the formation of the characteristic 4-methoxytryptophan residue of zelkovamycin, which is likely to originate from a tryptophan residue incorporated by domain A6.

Further analysis supported the existence of other undetected NRPS-associated enzymes in the coding sequences surrounding *zelA*–*C*, although their precise role in the biosynthesis and maturation of zelkovamycin could not be postulated with enough confidence. However, it is worth noting that ORFs 2697 (*zelF*) and 2687 (*zelG*), two biosynthetic genes located in close proximity of *zelA–E* (Fig 3B), both contain an A domain with predicted threonine selectivity and are flanked by additional biosynthetic genes coding for different types of enzymes. These independent modules could mediate the incorporation of Thr in the zelkovamycin precursor, which could then be further modified in *trans* or post-synthetically to yield the 2-amino-2-butenoic acid (i.e. dehydrobutyrine), the 2-aminobutyric acid or the 2-methyl dehydro threonine residues found in mature zelkovamycin [15].

Overall, this analysis supports the case for the biosynthetic gene cluster of zelkovamycin in *Actinomadura* sp. 32–07, in which the cluster is comprised of at least five adjacent genes, three of which are likely to be sufficient for the production of a complete zelkovamycin precursor. Thus, the zelkovamycin synthetase is postulated to consist of three core multimodular enzymes accounting for eight NRPS modules (Fig 3C). ZelA (2,719 aa) features the first three modules of the biosynthetic chain, with module 1 as the initiation module and module 3 being responsible for the incorporation of glycine and its methylation to *N*-methylglycine (i.e. sarcosine). The following three elongation modules are found in ZelB (3,567 aa), with the central one providing the cysteine selectivity and most (or all) the catalytic activities required for the formation of a thiazole ring with the upstream residue. ZelC (2,341 aa) encloses modules 7 and 8, the latter being responsible for the incorporation of glycine and acting as the termination module for the cyclisation and release of the completed peptide chain. Two additional enzymes, ZelE (a tryptophan 2,3-dioxygenase) and ZelD (an *O*-methyltransferase) are predicted to modify the Trp6 to afford a 4-methoxytryptophan residue.

**Fig 4:**
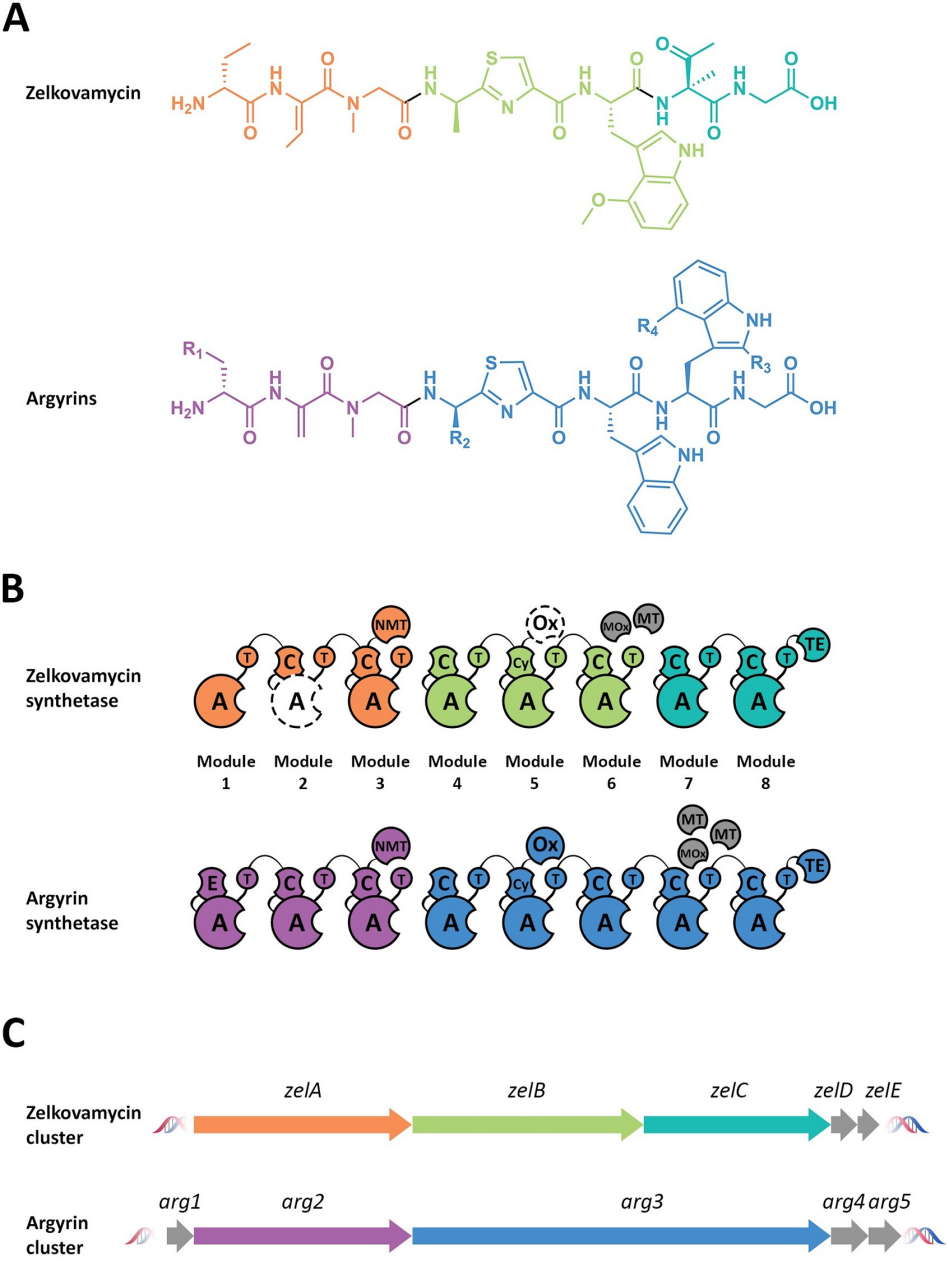
(A) Comparison between the linear structures of zelkovamycin and argyrins, (B) the NRPS systems behind their production and (C) the core biosynthetic genes coding for them. Genes, modules and amino acid residues of each compound are color-coded. The structure of the argyrin cluster and argyrin synthetase are adapted from Pogorevc et al. [60].

Very recently, the biosynthetic gene cluster of argyrins (a class of cyclic non-ribosomal peptides produced by *Archangium* and *Cystobacter* species) has been identified and characterised [60]. Due to the striking structural similarities between zelkovamycin and argyrins (Fig 4A), the NRPS system and, therefore, the gene composition and organisation of the biosynthetic cluster of argyrins can be expected to reflect those of zelkovamycin. Indeed, the comparison between the published argyrin synthetase and its characterised gene cluster further supported the present findings (Fig 4B and 4C). Additionally, the presence of an Ox domain fused to module 5 of argyrin synthetase corroborated the initial hypothesis that an oxidase activity may be provided by the corresponding module of the zelkovamycin synthetase. Furthermore, *arg4* and *arg5* (homologs of *zelD* and *zelE*, respectively) were identified to encode the *O*-methyltransferase and the tryptophan 2,3-dioxygenase responsible for the oxidation and methylation of the Trp7 to yield a 4-methoxytryptophan residue in argyrin, thus supporting the case for a similar function in the biosynthesis of zelkovamycin.

Following the completion of this work and during the preparation of this manuscript, the isolation of zelkovamycin and its four analogues zelkovamycin B-E from *Kitasatospora* sp. CPCC204717 [62] was reported. In this study, a putative biosynthetic cluster for the five peptides was proposed, which largely agrees with the model described in the present work. In particular, the *orf5*, *orf6* and *orf7* reported by Hao et al. supports the provisional role of *zelA*, *zelB*, and *zelC* as the main actors in the assembly of a zelkovamycin precursor. In further accordance with our observations, the presence of an Ox domain in module 5 has also been predicted in the zelkovamycin cluster (annotated Ox5) found in *Kitasatospora*, as well as the role of ZelF or ZelG as potential trans-acting standalone modules or domains providing the adenylating functionality missing from module 2.

Lastly, each of the five ZelA-ZelE proteins was queried with BLASTP against the non-redundant protein database at the NCBI (default parameters), collating the 100 top hits for each protein (S2 File). These 500 hits originated from 309 different database organisms, which TaxID numbers were extracted to build a Venn diagram (S2 Fig). Under these conditions, presently *Cystobacter* sp. SBCb004 (GenBank MK047651.1) is the only organism that can be found to encode significant homologues to all five proteins (S3 Fig) in the 32.9-kb argyrin biosynthetic gene cluster [60].

### Zelkovamycin production

**Fig 5:**
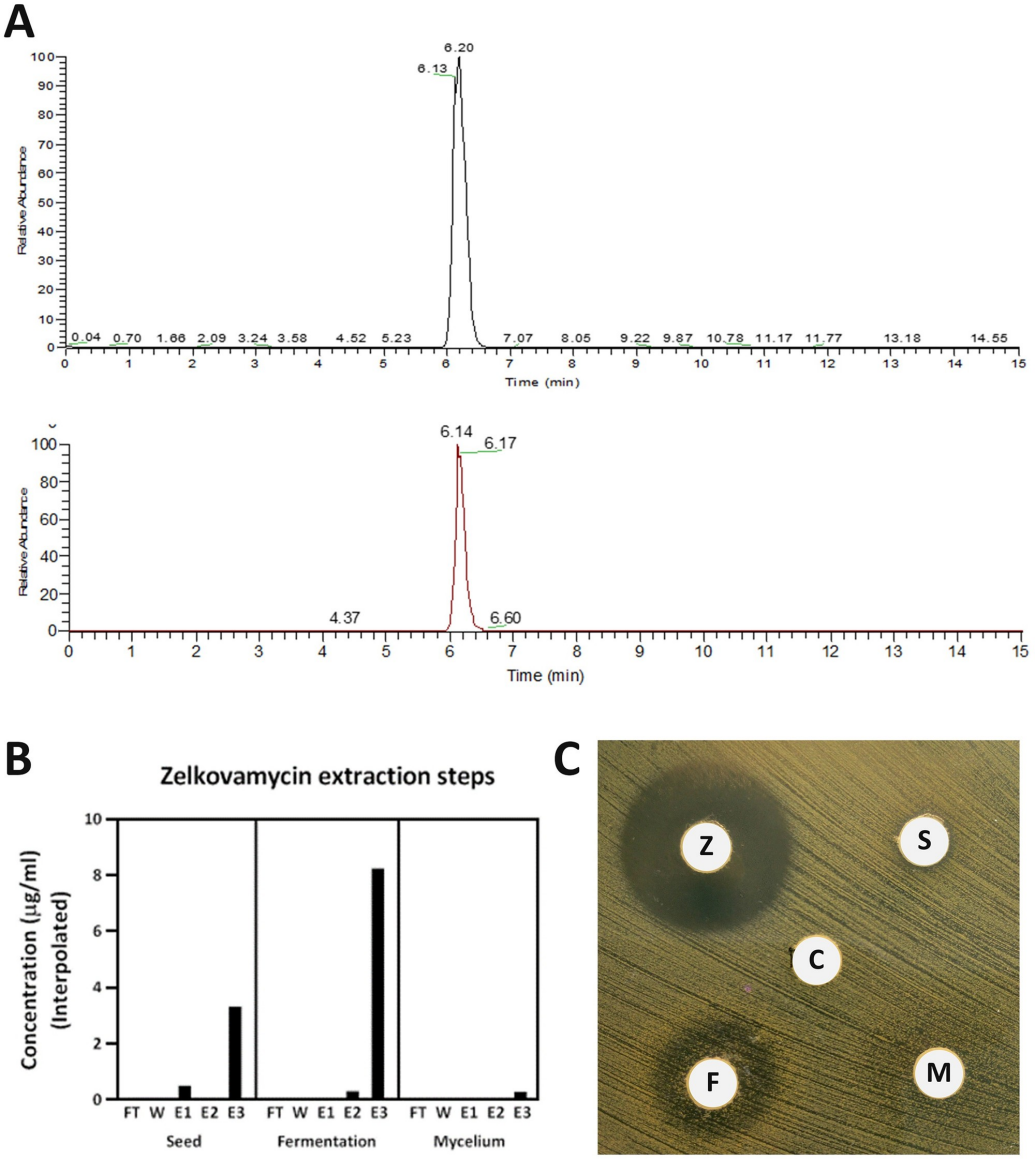
Detection, purification and antibiotic activity of zelkovamycin from *Actinomadura* sp. 32–07. (A) Extracted ion chromatograms of commercial zelkovamycin (top) and a representative example of an extracted sample (bottom). Zelkovamycin can be detected as a monodisperse peak eluted at 6.14 min. (B) Zelkovamycin extraction steps from the inoculum medium, the fermented medium and the harvested mycelium. The compound is present in both culture media and can be successfully extracted upon resin elution with 100% methanol. (C) Antibiotic sensitivity testing on *S*. *aureus* SH1000 using the most concentrated zelkovamycin extracts (E3) from each fraction. The lower concentration of antibiotic in the extracts obtained from the seed culture and the mycelium is reflected by their significantly lower, albeit detectable, inhibitory activity. FT: flow-through; W: water wash; E1–3: 20%, 50% and 100% MeOH elution, respectively; Z: zelkovamycin standard (1 mg/mL); S: seed culture extract; F: fermented broth extract; M: mycelium extract; C: 100% MeOH control.

Following 10 days of growth in fermentation broth, the spent medium and the mycelial biomass of *Actinomadura* sp. 32–07 were harvested, separated and subjected to solid-phase extraction using Diaion HP-20 polystyrene resin. In parallel, the starting seed medium used to inoculate the fermentative culture was also extracted with the same procedure. The resin from each sample was eluted with increasing concentrations of methanol in water, and all the eluates were analysed by LC-MS. In all cases, a monodisperse molecular ion peak (MH^+^ 780.314) could be detected and eluting at 6.14 min. These results were in line with the values obtained using a commercial sample of zelkovamycin (Fig 5A) and those found in the literature [12, 61], thus confirming that zelkovamycin was present in both the spent culture media and in the mycelium. However, the comparison between the area of the zelkovamycin peaks for each sample revealed that the fermentation medium contained approximately double the amount of zelkovamycin (8.3 μg/mL) than the seed medium (3.4 μg/mL), while only negligible quantities were present in the mycelium (Fig 5B). This result suggests that zelkovamycin is constitutively produced during the early stages of growth and actively released into the fermentation medium.

When tested against *Staphylococcus aureus* SH1000 by the disk diffusion method, the zelkovamycin extracts from the seed culture, the fermentation broth and the harvested mycelium all showed antimicrobial activity to variable extents, compatibly with the different concentration of antibiotic detected in each fraction (Fig 5C). Overall, the inhibitory effect was found to be in line with previous observations [12], indicating the zelkovamycin produced by *Actinomadura* sp. 32–07 retains its biological activity.

## Conclusions

*Actinomadura graeca* (grae.ca. N.L. fem. adj. *graeca* of or belonging to Greece, where the type strain was isolated) is an aerobic, Gram-positive, non-acid-alcohol-fast, non-motile actinomycete that forms extensively branched substrate mycelium and spore-bearing aerial hyphae. Long, flexuous series of subtle oval swellings carry an average of 10–20 small, spherical, smooth spores. Single round terminal swellings are also present sometimes. Growth on rich agar media is generally good and often appears brown, light brown or yellow, shifting to white or grey towards maturation. Melanoid pigments are not produced on ISP medium 6 and 7 or in any other tested media. Soluble colours, such as light yellow, orange and dark yellow, can be observed on ISP 1, GYEA and Tsukamura’s medium, respectively. Optimal growth conditions fall between 28–37 °C, pH 6.0–8.0 and 0–3% (w/v) of NaCl. D-glucose and sucrose are utilised as sole carbon sources on ISP medium 9, but not *myo*-inositol, D-mannitol, L-rhamnose, raffinose or cellulose. The microorganism degrades aesculin, arbutin, hypoxanthine, gelatine, starch, L-tyrosine, casein, Tween 20, Tween 40, Tween 60 and Tween 80 but not elastin, xanthine, xylan or tributyrin.

*Actinomadura graeca* is most sensitive to bacitracin, imipenem, linezolid, ticarcillin and clindamycin. DL-DAP is present in the cell wall, but it lacks mycolic acids. The predominant phospholipids are PI, DPG and PGL, rich in C16:0, C18:1 ω9c, 10-methyl-C18:0 and C16:1 ω9c fatty acids. The menaquinone system mainly consists of MK-9(H_6_), MK-9(H_4_) and MK-9(H_8_).

The results for most of the morphologic, physiological and chemotaxonomic tests performed on the isolate were in alignment with those expected from members of the genus *Actinomadura*. However, features like the lack of clearly visible spores, the presence of DL-DAP (rather than meso-DAP) and the absence of madurose (the diagnostic sugar, often found in trace amounts) stood out as the most salient differences.

The type strain 32-07^T^ (designated DSM 111581 and NCTC 14609 in the relevant culture collection) has been isolated from a soil sample in Santorini, Greece. Based on chemotaxonomic data and genomic, 16S rRNA and *rpoB*/*rpoC* gene sequence homologies, it is suggested that this strain represents a member of the genus *Actinomadura*. Nevertheless, its peculiar morphological, cultural and biochemical characteristics, as well as phylogenetic data, support the hypothesis that strain 32-07^T^ represents a novel species, for which the name *Actinomadura graeca* sp. nov. is proposed. The chromosome is made up of 8,944,773 bp with a G+C content of 72.36 mol%.

Significantly, the new species has been confirmed as a producer of the macrocyclic peptide antibiotic zelkovamycin. The antibiotic could be extracted from a liquid culture of the strain by incubation with a polystyrene resin and it is maximally eluted with 100% methanol. When tested against *S*. *aureus* SH1000, the extracted peptide was found to retain the antibacterial activity highlighted in previous studies [12, 61]. During the preparation of this manuscript, Hao et al. reported that zelkovamycin displayed potent antimicrobial activity against both *S*. *aureus* ATCC 29213 and *S*. *epidermidis* ATCC 12228 [62].

Most of the biosynthetic genes coding for the NRPS machinery behind the production of zelkovamycin have been uncovered in this study. The provisional model consists of three core biosynthetic genes (*zelA–C*) coding for a total of eight NRPS modules, the majority of which contain the expected C, A and T domains required to direct the biosynthesis of a complete zelkovamycin precursor. Two additional genes (*zelD–E*) are also proposed to be part of the biosynthetic gene cluster, as they encode for a stand-alone tryptophan 2,3-dioxygenase and an *O*-methyltransferase, and are likely involved in the production of a key residue, 4-methoxytrptophan in zelkovamycin. The comparison of the putative *zel* cluster with the recently published cluster for the structurally related argyrins [60] revealed several similarities that corroborate these conclusions. Nevertheless, further biochemical data is needed to validate the present findings and unambiguously identify the remaining genes involved in the biosynthesis of zelkovamycin.

In conclusion, the data hereby provided represents the first instance of a species outside the genus *Streptomyces* being reported as a source of zelkovamycin and paves the way towards the exploitation of the biosynthetic potential of this microorganism.

## Supporting information

S1 File Initial isolation of *Actinomadura graeca* 32–07. (PDF)

S2 File Each of the five ZelA-ZelE proteins was queried with BLASTP against the non-redundant protein database at the NCBI, and the resulting 100 top hits for each protein are listed. (XLSX)

S1 Fig Maximum likelihood tree generated from the RNA polymerase subunit genes *rpoB/rpoC* showing the relationship between *Actinomadura* sp. 32–07 and the type strains of species in the genus *Actinomadura*. (TIF)

S2 Fig Venn diagram representing the conservation of the ZelA-ZelE proteins among diverse GenBank taxa. The five proteins were individually queried with BLASTP against the NCBI non-redundant protein database (default parameters) and unique TaxIDs of the first 100 hits for each protein (309 TaxIDs in total) were extracted to build the diagram. Under these conditions, only two organisms were found to encode significant homologues to these five proteins: *Actinomadura* 32–7 (source of the sequences) and *Cystobacter* sp. SBCb004.
(TIF)

S3 Fig Alignment and homologies of the *zelABCDE* and *arg12345* clusters of *Actinomadura* 32–7 and *Cystobacter* sp. SBCb004, respectively, at the protein levels. Overall, the ZelABCDE proteins are in average 51% identical and 65% similar to the Arg2345 proteins over 98% of their cumulated length, with a significant homologue of Arg1 (radical SAM-dependent methyltransferase) not being found to be encoded in the genome of *Actinomadura* 32–7. i: percent identity, s: percent similarity.
(TIF)
