# Supplementary material for: Actinomadura graeca sp. nov.: A novel producer of the macrocyclic antibiotic zelkovamycin
Source: PLoS One. 2021 Nov 30;16(11):e0260413. doi: 10.1371/journal.pone.0260413 (PMC8631618; doi:10.1371/journal.pone.0260413)
Supplement: S1 File — (PDF) [file pone.0260413.s001.pdf]

Initial isolation of *Actinomadura graeca* 32-07

The strain was initially isolated from a soil sample collected in Santorini, Greece by using the Andersen method (S1). The isolation media was Humic acid-vitamin agar according to Hayakawa (S2); the agar plate was incubated for 3 weeks at 28 °C and the strain was selected by morphology and microscopic observation.

References

(S1) Anderson AA. New sampler for the collection, sizing, and enumeration of viable airborne particles. *J Bacteriol.* 1958;76(5):471-84.

(S2) Hayakawa M, Nonomura, H. Humic acid-vitamin agar, a new medium for the selective isolation of soil actinomycetes. *J Ferment Technol.* 1987;65(5), 501-09.
