# Supplementary figures and images for: Actinomadura graeca sp. nov.: A novel producer of the macrocyclic antibiotic zelkovamycin
Source: PLoS One. 2021 Nov 30;16(11):e0260413. doi: 10.1371/journal.pone.0260413 (PMC8631618; doi:10.1371/journal.pone.0260413)

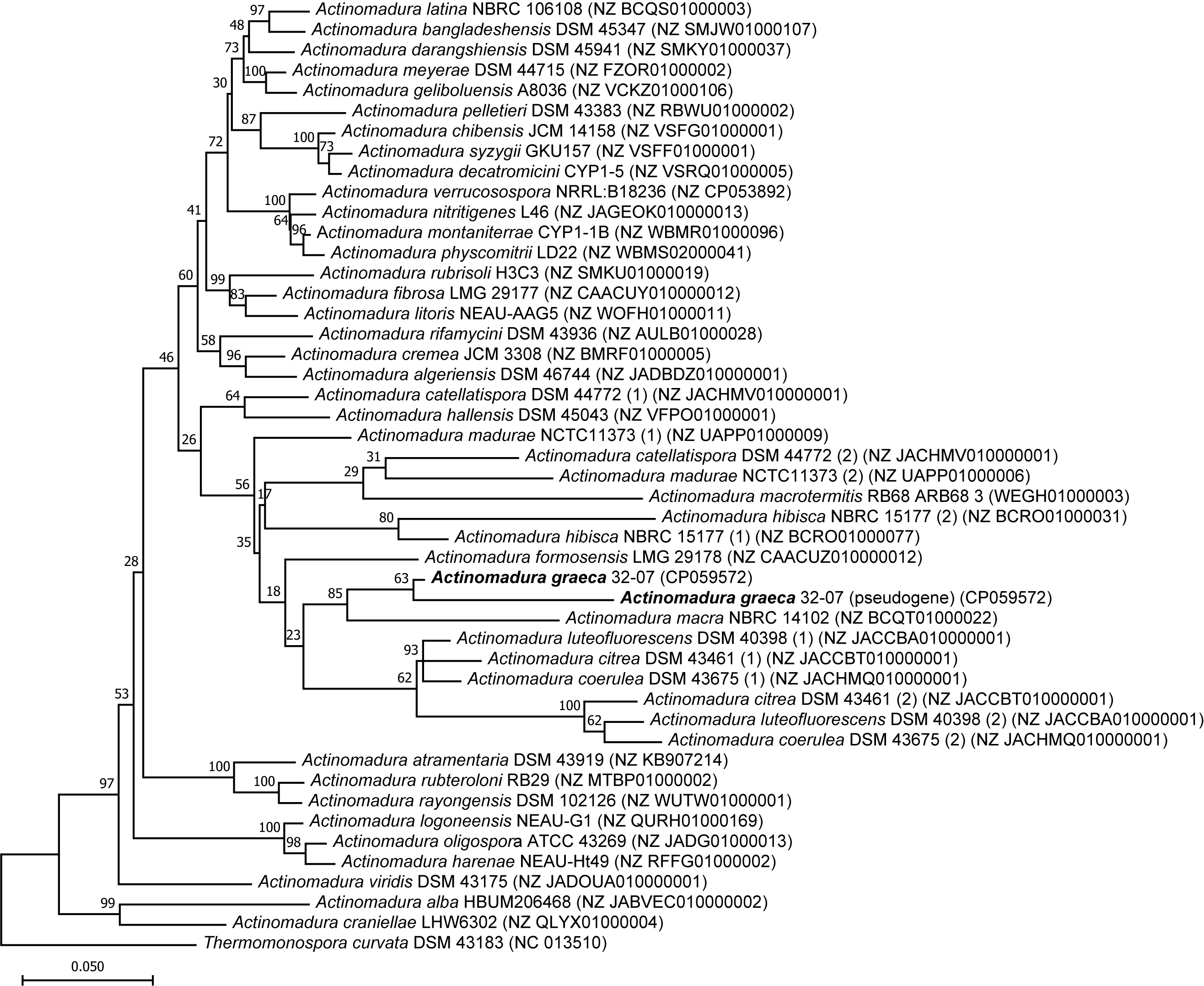

Supplement: S1 Fig — (TIF) [file pone.0260413.s003.tif]

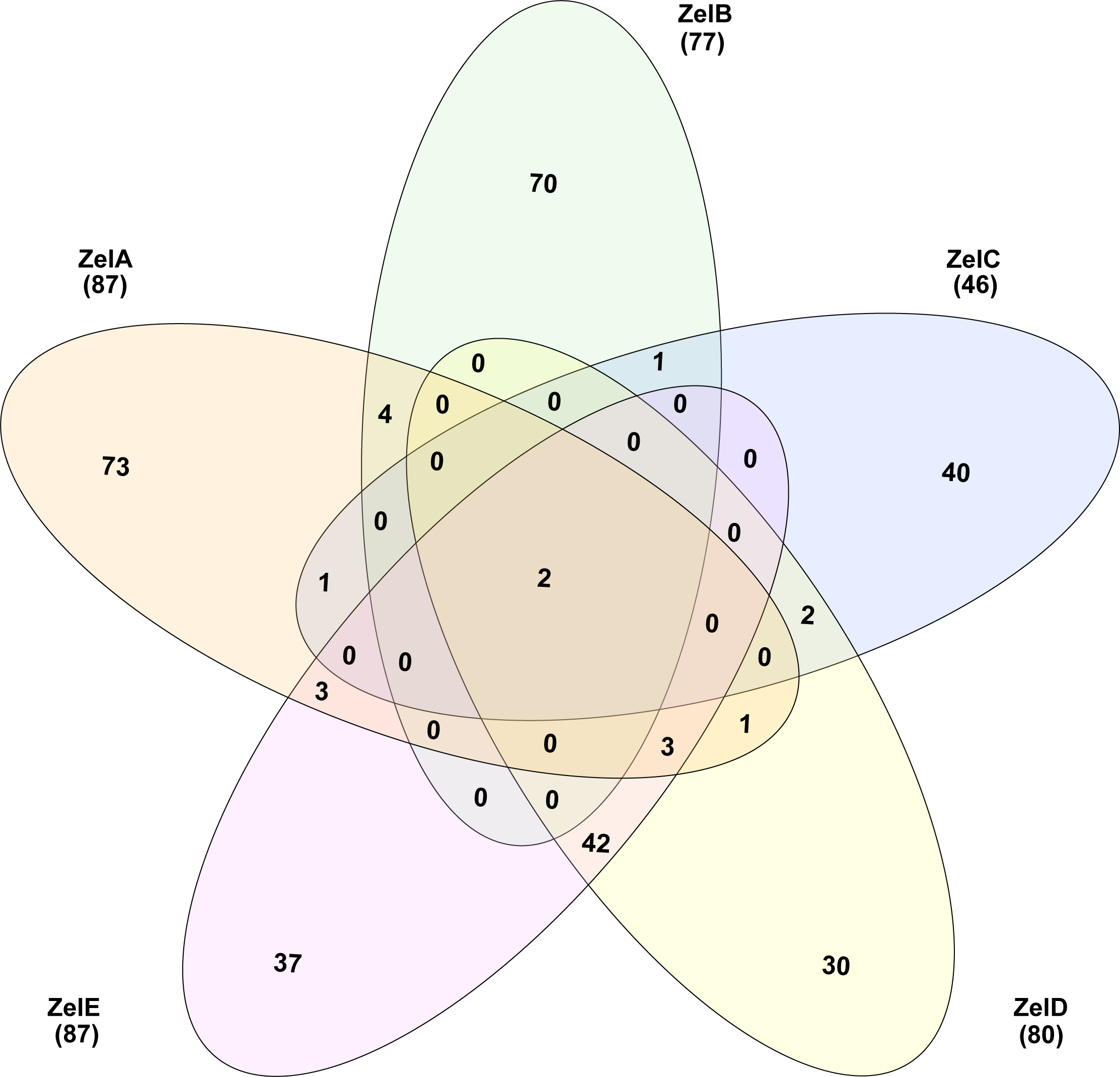

Supplement: S2 Fig — The five proteins were individually queried with BLASTP against the NCBI non-redundant protein database (default parameters) and unique TaxIDs of the first 100 hits for each protein (309 TaxIDs in total) were extracted to build the diagram. Under these conditions, only two organisms were found to encode significant homologues to these five proteins: Actinomadura 32–7 (source of the sequences) and Cystobacter sp. SBCb004. (TIF) [file pone.0260413.s004.tif]

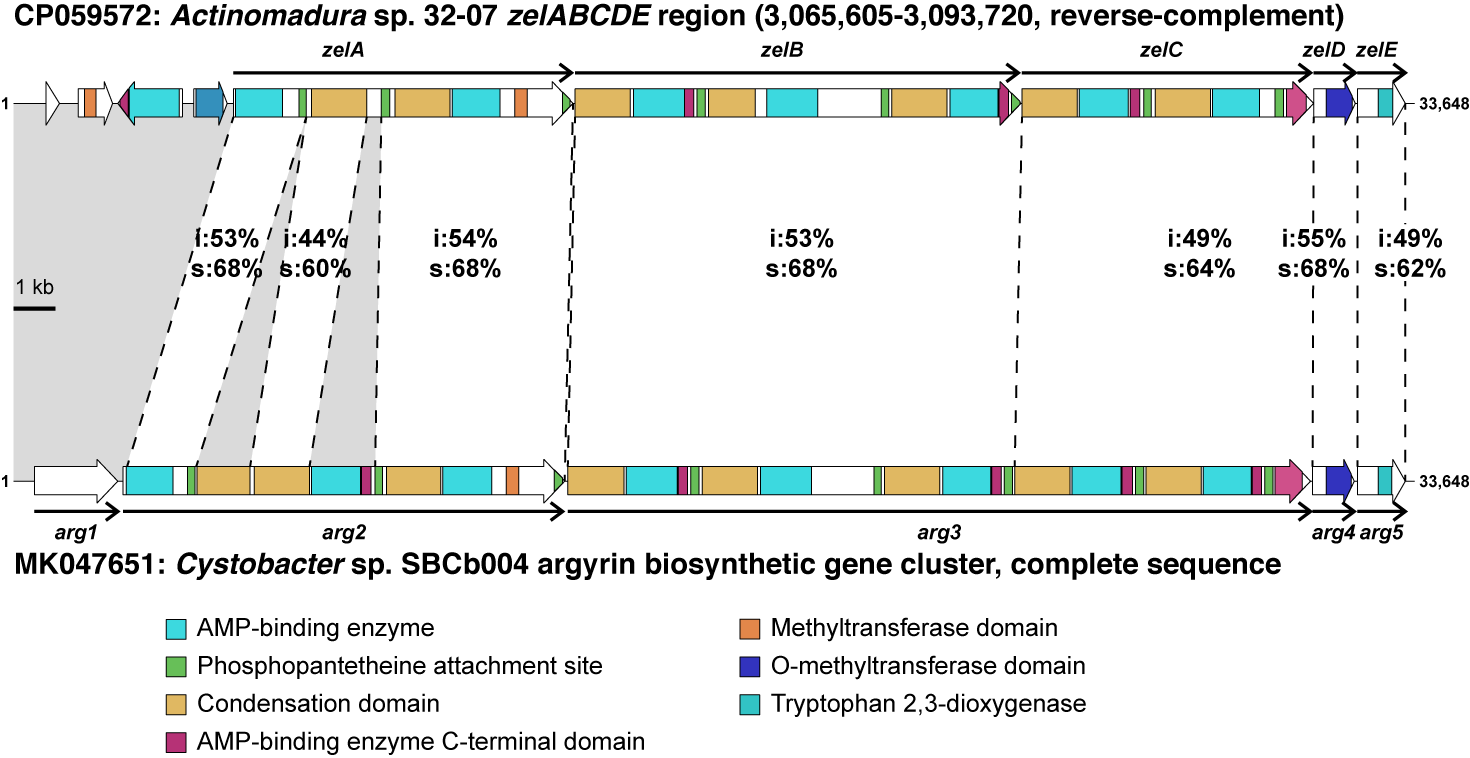

Supplement: S3 Fig — Overall, the ZelABCDE proteins are in average 51% identical and 65% similar to the Arg2345 proteins over 98% of their cumulated length, with a significant homologue of Arg1 (radical SAM-dependent methyltransferase) not being found to be encoded in the genome of Actinomadura 32–7. i: percent identity, s: percent similarity. (TIF) [file pone.0260413.s005.tif]
